# Ferric Carboxymaltose in the treatment of chemotherapy-induced anaemia: an effective, safe and cost- sparing alternative to blood transfusion

**DOI:** 10.1038/s41598-019-56999-3

**Published:** 2019-12-31

**Authors:** Joana Marinho, Inês Leão, Sandra Custódio, Enrique Dias, António Moreira Pinto, Telma Costa, Andreia Capela, Margarida Dias, Henrique Coelho, Ângela Cunha, Ana Macedo, Anabela Amarelo, Ana Joaquim

**Affiliations:** 1 Centro Hospitalar Vila Nova de Gaia/Espinho, Medical Oncology Department, Vila Nova de Gaia, Portugal; 2 Centro Hospitalar Vila Nova de Gaia/Espinho, Pneumology Department, Vila Nova de Gaia, Portugal; 3 Centro Hospitalar Vila Nova de Gaia/Espinho, Clinical Haematology Department, Vila Nova de Gaia, Portugal; 4 Centro Hospitalar Vila Nova de Gaia/Espinho, Imunohemotherapy Department, Vila Nova de Gaia, Portugal; 5 Keypoint - Consultoria Científica, Algés, Portugal

**Keywords:** Health care, Oncology

## Abstract

Anaemia is highly prevalent in cancer patients, adversely affects quality of life and impacts survival. The pathogenesis is multifactorial, with iron deficiency being a major and potentially treatable contributor. This study aimed to assess the effectiveness and economic impact of ferric carboxymaltose in chemotherapy-induced anaemia. This prospective cohort study between 2015–2016 of chemotherapy-treated patients for solid tumours, grade ≥2 anaemia and iron deficiency evaluated hematopoietic response four weeks after ferric carboxymaltose treatment. Transfusion rate of all cancer patients treated at our ambulatory unit during the two-year study period (2015–2016) was compared to a retrospective cohort (2013–2014) who received blood transfusion only. Between 2015–2016, 99 patients were included and treated with ferric carboxymaltose, the majority of whom (n = 81) had relative iron deficiency. Mean haemoglobin concentrations improved from 9.2 [6.7–10.8] g/dL to 10.6 [7.8–14.2] g/dL four weeks after treatment. A 26% reduction in the transfusion rate was observed from control retrospective to the prospective study group including ferric carboxymaltose treated patients [relative risk 0.74 (95% CI:0.66–0.83)]. The cost analysis showed a benefit for the use of ferric carboxymaltose in chemotherapy-induced anaemia. This study shows that ferric carboxymaltose is an effective, cost-saving support treatment, reducing the need for allogeneic transfusions saving blood units which are a limited resource.

## Introduction

Quality of life should be a priority in the management of cancer patients. As one of the most common problems reported at diagnosis and during cancer treatment, anaemia is a major concern in any Oncologist’s daily practice. The incidence of anaemia in cancer patients reported in literature varies significantly, ranging from 20% to 60% at the time of cancer diagnosis and reaching 60–90% during cancer treatments^1–4^. Anaemia has a negative impact on the quality of life of cancer patients. It is associated with poor performance status, fatigue, and may also jeopardize adherence to treatment, affecting therapeutic results, hospital stay and even survival^4–6^. According to the World Health Organization (WHO), anaemia is defined as haemoglobin (Hb) levels <12.0 g/dL in women and <13.0 g/dL in men^7^. The National Cancer Institute subdivides anaemia into different grades: mild – grade 1 (10 g/dl—normal), moderate – grade 2 (8–<10 g/dl), severe – grade 3 (6.5–<8 g/dl) and life threatening (<6.5 g/dl) anaemia^8^.

In cancer, anaemia has a multifactorial etiology. Iron deficiency (ID) is one of the underlying causes as its prevalence varies from 32 to 60%^6,9,10^. Anaemia may be attributed to absolute ID that can result from chronic blood loss due to gastrointestinal and gynaecological malignancies or surgery. Although less frequent, it can also derive from nutritional deficiencies due to cancer-induced anorexia, as well as reduced iron absorption due to gastrectomy, or the use of proton pump inhibitors, taken approximately by 20% of cancer patients^11,12^. The production of cytokines that leads to chronic inflammation and iron sequestration, and the myelosuppressive effects of chemotherapy or metastatic infiltration of the bone marrow limiting erythropoiesis are also contributing factors^4,10,13,14^. A variety of factors have been reported to be predictive of cancer and chemotherapy-associated anaemia: recent anticancer therapy, old age, poor performance status, advanced stage of the disease and particular tumour location (pancreatic, colorectal and lung cancer)^6^.

ID can be classified as absolute when iron stores are depleted, mainly due to bleeding, which corresponds to between 7 and 42% of all cases^10,15^, or functional (29–46% of all cases), when iron reserves are normal or increased but sequestered inside macrophages and enterocytes^10,15,16^. In the latter case reduction of iron availability for erythropoiesis is observed leading to anaemia.

Considering absolute ID, while in normal individuals a serum ferritin level of <30 ng/mL is virtually diagnostic, in cancer patients a higher ferritin cut-off (<100 ng/mL) appears more reliable, due to the chronic inflammation status. In functional ID, the guidelines available recommend testing both serum ferritin and transferrin saturation (TSAT). European Society for Medical Oncology (ESMO) guidelines considers functional ID when TSAT is <20%^17^, whereas The National Comprehensive Cancer Network (NCCN) guidelines targets ferritin level between 30 ng/mL and 500 ng/mL and a TSAT level <50%^8^.

Once other causes of anaemia are excluded, most patients with cancer and chemotherapy-induced anaemia (CIA) are treated with red blood cell (RBC) transfusion and/or iron supplement as monotherapy or in association to erythropoiesis-stimulating agents (ESA)^8,17,18^. RBC transfusions increase the risk of thrombotic events, may decrease survival^19^ and must be reserved for patients with severe anaemia symptoms in need of rapid Hb improvement. Safety issues regarding ESA’s link to decreased survival or increased disease progression were raised in the past. However recent data from a meta-analysis and prospective trials did not reveal links to tumour progression or reduced survival prospects^20,21^. Venous thromboembolic events are a known risk of ESA use in cancer patients^22^. Therefore, anaemia treatment guidelines do not recommend transfusions and suggest minimization of ESA dosage^8,17^. Some guidelines go as far as limiting their use to patients whose cancer treatment is not curative in intent^18^. Studies showing that intravenous (IV) iron (with or without concomitant ESA therapy) can improve Hb levels and reduce transfusion requirements in cancer patients support these goals^23–25^.

There are a number of IV iron formulations in the market^26^. In Portugal, four are available: iron sucrose, low molecular weight iron dextran (LMW-ID), iron isomaltoside and ferric carboxymaltose (FCM), all studied in CIA (reviewed in^27^). Because of its stable complex which allows for a slow and prolonged iron release, FCM enables administration of 1000 mg of iron in a 15-minute infusion^28,29^. Moreover, as reported by several authors in a variety of anaemia backgrounds, FCM is safe and effective providing a rapid correction of Hb and serum ferritin levels in iron-deficient patients^30^.

Therefore, the main objective of this study was to assess the effectiveness of FCM in the treatment of CIA.

## Results

Between 2015 and 2016, 99 patients with at least grade 2 anaemia (Hb < 10 g/dl) and iron deficiency (defined as ferritin <800 ng/mL and TSAT <50%)^8^ were included in the study and treated with FCM infusions according to body weight as described in the methods section (a total of 1500 mg if 35 Kg to <70 Kg or 2000 mg if ≥70 Kg). Median age was 66 years [31–84 years] and 49.5% (n = 49) were male (Table 1). The most frequent type of tumours were gastrointestinal (44.4%, n = 44) and breast (21.2%, n = 21), and 39.4% (n = 39) had an advanced stage disease (stage IV) at diagnosis (Table 1).

**Table 1:** Baseline patient characteristics (demographics and disease characteristics).

Variables	Ferric Carboxymaltose group
Age (years), median, IQR^a^	66 ± 16
**Gender**, **n (%)**	
Male	49 (49.5)
Female	50 (50.5)
**Cancer type**, **n (%)**	
Colorectal	22 (22.2)
Gastric	22 (22.2)
Breast	21 (21.2)
Pancreas	11 (11.1)
Gynaecological	7 (7.1)
Lung	4 (4.1)
Others^b^	12 (12.1)
**Disease stage at diagnosis, n (%)**	
I-III	60 (60.6)
IV	39 (39.4)
**Intention treatment at inclusion, n (%)**	
Curative	45 (45.5)
Palliative	54 (54.5)
**Ongoing chemotherapy at inclusion, n (%)**	94 (94.9%)
**Iron Deficiency, n (%)**	
Absolute	18 (18.2)
Relative	81 (81.8)

^a^Abbreviations: IQR – interquartile range.

^b^Others: head and neck, cholangiocarcinoma, oesophagus, occult primary tumour, urothelial.

The majority of patients received cytotoxic chemotherapy (94.9%, n = 94) and 5 (5.1%) participants were not receiving anti-cancer treatment at the start of the study. Treatment intent was curative in 45 patients. During the treatment phase, the median [Interquartile Range (IQR)] total dose of iron received was 1500 (±500) mg. The majority of participants (74.7%) received two infusions.

**Figure 1:**
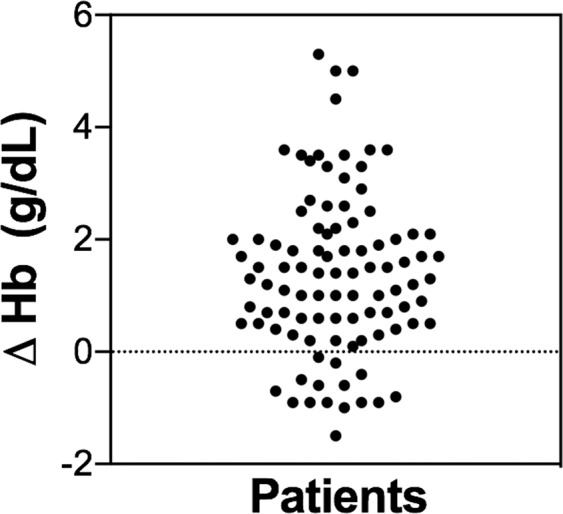
Haemoglobin level variation between baseline (pre-treatment) levels and four weeks after ferric carboxymaltose administration. Each dot represents a value relative to a patient.

Iron status was assessed before study enrolment, and the majority (81.8%, n = 81) had functional ID (Table 1). Baseline haematological parameters are described in Table 2 and were typical of a cancer population. The baseline mean Hb concentration was 9.2 [6.7–10.8] g/dL. Four weeks after the last FCM course of treatment Hb increased in treated patients (*versus* baseline) on average to 10.6 [7.8–14.2] g/dL (Table 2). These results showed a statistically significant increase in Hb concentrations after FCM administration (p < 0.0001). An increment in Hb concentration from baseline was observed in 84 patients (84.8%) ranging from 0.1 to 5.3 g/dL (Fig. 1). In 23.2% of participants those increments were higher than 2 g/dL (Fig. 1).

**Table 2:** Haematological parameters at study inclusion and increase in Hb from baseline until the end of study period.

Variables	Ferric Carboxymaltose group
**Baseline Hb (g/dL)**	
Mean, SD^a^	9.2 ± 0.8
Min-Max^a^	6.7–10.8
**Post-treatment Hb**^**b**^ **(g/dL)**	
Mean, SD^a^	10.6 ± 1.3
Min-Max^a^	7.8–14.2
**Δ Hb (g/dL)**	
Mean, SD	1.37 ± 1.44
Max-Min	(−1.5)–5.3
*p value* (Baseline vs post-treatment Hb)	<0.0001
**Baseline ferritin (ng/mL)**	
Mean, SD^a^	255 ± 222
Min-Max^a^	4.0–790
**Baseline TSAT (%)**	
Mean, SD^a^	15.5 ± 9.4
Min-Max^a^	3.0–38.4

^a^Abbreviations: SD – standard deviation; Min – Minimum; Max – Maximum; TSAT – Transferrin saturation.

^b^Hb value at follow-up visit during week 4.

^c^Data were analysed by paired t-test. The significance level was considered p < 0.05.

Eleven patients (11.1%) received RBC transfusion within four weeks after the first FCM administration, and 26 (26.3%) after week four. RBC transfusions did not influence mean Hb concentrations achieved, as the mean post treatment Hb in the 88 patients treated with FCM-only was similar: Hb 10.7 [8.2–14.2] g/dL *vs* post-treatment Hb 10.6 [7.8–14.2] g/dL in all 99 FCM-treated patients.

A total of 319 vials of 500 mg of FCM were used in the study. Treatments were well tolerated, and neither hypersensitivity reactions nor other severe drug-related adverse events (immediate or delayed) occurred.

With regard to the secondary endpoint transfusion rate, we compared a cohort that comprises 99 patients treated with FCM and all patients subjected to RBC transfusion in the years 2015–2016 (prospective cohort), to a historical control group from two years before (2013–2014). The control group had received RBC transfusion as the only treatment for anaemia.

During the study period (2015–2016), a total of 13221 chemotherapy sessions were performed in 1811 patients, and a total of 517 blood units were used, representing a transfusion rate (number of RBC units per chemotherapy session) of 3.9% (Table 3). In the previous two years (control group: 2013–2014), 657 blood units were used in 12322 chemotherapy sessions, totalling a higher transfusion rate (5.3%) (Table 3). These results show a 26% reduction in the transfusion rate between the control and the study group including FCM-treated patients, with a relative risk of 0.74 (95% CI: 0.66–0.83, p < 0.0001).

**Table 3:** Transfusions and FCM infusions.

	Retrospective control group (2013–2014)	Prospective group (2015–2016)
Patients, n	1732	1811
ChT^a^ cycles, n	12322	13221
Number of transfusions, n	194	189
RBC^a^ units, n	657	517
Patients treated with FCM, n	0	99
Total FCM vials, n	0	319
% of patients with transfusion	11.2	10.4
Transfusion rate^b^ (%)	5.3	**3**.**9**

^a^Abbreviations: ChT – Chemotherapy; RBC – red blood cell; FCM – ferric carboxymaltose.

^b^Transfusion rate was calculated as the following: number of RBC units per chemotherapy treatment.

The unitary costs elements used for the analysis of FCM economic impact are detailed in Table 4. The results show a benefit per patient (−2.00 €) and chemotherapy cycle (−0.56 €), of using FCM treatment in CIA relative to RBC transfusion (Tables 5 and 6). Even though the direct total cost savings might appear low, the indirect cost savings are important, allowing a reduction in RBC units which are a crucial and limited resource.

**Table 4:** Cost elements.

Ferric carboxymaltose infusion (500–1000 mg/session)	
**Intravenous iron**	
FCM acquisition cost^a^	€ 95.38/500 mg
FCM administration cost^b^	€ 12.14/infusion
**Red Blood Cell Transfusion**	
RBC acquisition cost^c^	€ 164/unit
Pre-transfusion tests costs^d^	€ 28
RBC administration cost^b^	€ 20.90

Abbreviations: RBC – red blood cell; FCM – ferric carboxymaltose.^a^Manufacturer’s selling price; ^b^Official price for Portuguese Health System; ^c^Portuguese Health System tariff for acquisition of a red blood cell unit; ^d^Includes two patients’ blood group assessments, one screening for irregular antibodies, one direct coombs test and compatibility tests.

**Table 5:** Global treatment costs (red blood cell transfusions + Ferric carboxymaltose treatment).

	Retrospective Control group	Prospective group
Transfusions^a^	139 875.30 €	110 069.30 €
Ferric Carboxymaltose^b^	—	32 562.40 €
Total costs	139 875.30 €	142 631.70 €

^a^Transfusion costs were calculated multiplying RBC units for the unitary cost (RBC acquisition cost + pre-transfusion tests costs + administration costs) – see Tables 3 and 4.

^b^FCM costs were calculated multiplying the number of vials (see Table 3) for the FCM acquisition cost, adding the cost of FCM administration taking into consideration that the infusion price is for 1000 mg/session.

**Table 6:** Cost-effectiveness of Ferric Carboxymaltose treatment (per patient and chemotherapy cycle).

	Retrospective Control group	Prospective group
Total cost per patient	80.76 €	78.76 €
Total cost per ChT cycle	11.35 €	10.79 €
Incremental cost per patient	—	**− 2**.**00 €**
Incremental cost per ChT cycle	----	**− 0**.**56 €**

Costs were calculated considering the total number of patients and chemotherapy cycles described in Table 3.

## Discussion

IV iron supplementation is widely used for the treatment of chronic iron deficiency anaemia. FCM was shown to be an effective treatment for anaemic patients with chronic kidney disease undergoing haemodialysis, chronic heart failure, post-partum anaemia or inflammatory bowel disease^30^. However, its usage in cancer patients is limited^23,24^. In this study, we showed that the treatment with FCM in cancer patients undergoing chemotherapy (with a grade ≥2 anaemia and iron deficiency) was effective in 85% of cases, improving the mean Hb levels of anaemic cancer patients. These observations are in line with the results published by *Steinmetz et al*. and *Toledano et al*., where the supplementation with FCM only, in anaemic cancer patients with absolute iron deficiency under chemotherapy, led to a substantial and sustained increase in Hb levels, suggesting a role for IV iron as first-line treatment for CIA^23,24^.

Currently, the treatment of anaemia in cancer patients consists of RBC transfusions and use of ESAs with or without iron supplementation^8,17^. Although ESAs are useful, ASCO guidelines limits their use to palliative intent^18^, and NCCN recommends its use only in patients receiving myelosuppressive chemotherapy or undergoing palliative treatment^8^.

At recommended doses, IV iron is well tolerated, particularly when compared with oral iron. FCM was a safe treatment as no severe drug-related adverse events were reported in this study.

Compared to other IV iron formulations, the incidence of transient hypophosphatemia with FCM is fairly high as reported in two retrospective studies^31,32^, and a recent prospective trial^33^, although no serious adverse events due to low phosphate values were seen^28,31,32^. Therefore, serum phosphate levels should be determined in all FCM treated subjects.

FCM has been prospectively compared to other IV iron formulations and showed similar safety and efficacy levels. As showed in a systematic review and meta-analysis of 21 randomized controlled trials by *Rognoni et al*., FCM resulted in a higher increase of serum ferritin levels in comparison to ferric gluconate and showed a high safety profile^30^, consistent with the other formulations able to be administered as a total iron dose infusion (LMW-ID, iron isomaltoside and ferumoxytol)^26^.

The main advantage is the administration of a single total dose, which decreases the risk of reactions to multiple courses of treatment. In addition to being a convenient option for the patient, it also reduces the number of interventions from medical staff and reduces ambulatory clinic booking hours, which contributes to a reduction in costs.

Additionally, our results showed a significant reduction in the percentage of cancer treatments that needed transfusion support in the group that included patients treated with FCM, reducing the need of allogeneic transfusion, and therefore saving RBC units.

For all these reasons, supplementation with IV iron, stands as an attractive therapeutic option for treatment of anaemic cancer patients with iron deficiency.

The cost reduction analysis was also favourable, as a direct cost saving was achieved in the FCM treatment group, due to the reduced need for RBC transfusions. More importantly, a number of indirect cost savings were achieved, namely in terms of time spent at ambulatory clinic and number of RBC units saved. From the patient’s perspective, those savings translate into fewer hospital visits and work absences, reduced transportation costs to name a few.

FCM was already shown to have reduced direct and indirect costs of hospitalization compared with iron sucrose or oral iron^34^. Cost reduction analysis also favours FCM over iron sucrose for the ambulatory treatment of iron deficiency^35^. Two studies, from Italy and Brazil, showed that FCM might be a cost-saving option for their health care systems when compared to ferric gluconate or iron sucrose in the treatment of iron-deficient patients^36,37^. With regards to the treatment of CIA, no data is available.

This topic is of major importance in clinical practice as anaemia is highly prevalent among cancer patients and its treatment might have a significant impact on survival and quality of life^5,6^. Iron deficiency is associated with impaired physical function, weakness and fatigue even in the absence of anaemia^9^.

One potential limitation arising from the design of our study is the lack of long term follow-up data on Hb values or patient-reported outcomes, which would have been helpful in assessing the clinical significance of our findings. Cost issues needs to be considered on a country level, as significant price differences for FCM between Europe and the United States are present.

Future studies may assess the benefit of this treatment in patients without anaemia, despite the documented iron deficiency, as it may not only prevent the occurrence of anaemia, but also improve symptoms of iron deficiency.

## Methods

### Patients and study design

A prospective cohort study was performed in a central Portuguese hospital (Centro Hospitalar Vila Nova de Gaia/Espinho) from 1^st^ January 2015 to 31^st^ December 2016. The study was reviewed and approved by the ethics committee of this hospital. All data was collected from electronic medical records. Data regarding the group which includes patients treated with FCM was collected prospectively. Data regarding the control group was collected retrospectively at baseline, for the period between the 1^st^ January 2013 and 31^st^ December 2014. The eligible population consisted of adults (≥18 years old) with a malignant solid tumour and diagnosed with at least grade 2 anaemia (defined by Common Terminology for Adverse Events v.5.0 as haemoglobin inferior to 10 g/dL^8^) and iron deficiency (defined as ferritin level <800 ng/mL and TSAT <50%)^8^. All were undergoing anti-cancer treatment and signed an informed consent. Subjects who received more than 50 units of blood or patients treated with ESAs were excluded from the study. Cancer staging was set in accordance to the TNM Classification (AJCC 7^th^ edition).

At the start of the study, all patients were analysed for ferritin levels, transferrin saturation, vitamin B12 and folic acid status. The group treated with FCM consisted of subjects who initiated treatment with FCM (Ferinject, Vifor Pharma, Glattbrugg, Switzerland) between the 1^st^ of January 2015 and 31^st^ of December 2016. Treatment was prescribed when ferritin was lower than 800 ng/mL and TSAT was inferior to 50%. If patient body weight was 35 Kg to <70 Kg the dosage recommended was 1000 mg+ 500 mg (with at least one week interval between administrations). If patient weight was greater than 70 Kg the dosage recommended was 1000 mg + 1000 mg (with at least one week interval between administrations). Patients were examined during the first half hour post-administration for any possible side effects such as pain at the site of injection, flushing, allergic reactions, headache, dizziness, nausea, vomiting and a feeling of heaviness in the head. Haemoglobin levels were assessed four weeks after the last treatment dosage. The main aim of this study was to assess the effectiveness of FCM in the treatment of CIA in patients diagnosed with solid tumours. In order to do so, we analysed the hematopoietic response, defined as a positive haemoglobin variation 4 weeks after the post-carboxymaltose treatment.

This study also aimed to compare the transfusion rate, defined as the number of RBC units per chemotherapy treatment, between the cohort that included patients treated with FCM and those of a historical control group. The control group fulfilled the same inclusion criteria but had received RBC transfusions as the only form of treatment for anaemia.

A safety endpoint was to assess the number of adverse events during FCM treatment. The authors also analysed the economic impact of FCM protocol. Informed consent was obtained from all individual participants prior to the start of the study inclusion.

### Cost and statistical analysis

Categorical variables are presented as frequencies and percentages, and continuous variables as means and standard deviations, or medians and interquartile range (IQR) for variables with skewed distributions. Normal distribution was checked using skewness and kurtosis. A dependent t-test for paired samples was used for analysis of Hb variation over time (Hb at baseline vs Hb 4 weeks after treatment). Analysis of the results was performed using SPSS statistical software, version 22.With regards to the cost analysis (Table 4), for the control group transfusion costs were calculated multiplying RBC units for the unitary cost (RBC acquisition cost + pre-transfusion tests costs + administration costs). Costs for the FCM group were calculated taking into account the costs of FCM vials, costs of administration (taking into consideration that the infusion price is for 1000 mg/session) and the cost of RBC transfusions as calculated for control group. Additionally, the economic impact per patient and chemotherapy cycle was calculated. The costs were provided by the hospital and Portuguese health system tariffs. We did not include the costs of laboratory tests and investigations (to diagnose the presence and cause of anaemia); follow-up appointments (assumed to be the same in both study arms); surveillance costs, in the post-transfusion and post-FCM treatment; societal costs (e.g., loss of working hours).

### Compliance with ethical standards

The study was reviewed and approved by the ethics committee of Centro Hospitalar Vila Nova de Gaia/Espinho, where the study was conducted and was approved by unanimity on the 29^th^ December 2014. All procedures performed in studies involving human participants were in accordance with the ethical standards of the institutional and/or national research committee and with the 1964 Helsinki declaration and its later amendments or comparable ethical standards.
